# Exploring Applications and Preparation Techniques for Cellulose Hydrogels: A Comprehensive Review

**DOI:** 10.3390/gels10060365

**Published:** 2024-05-25

**Authors:** Yanjin Tang, Zhenxing Fang, Hoo-Jeong Lee

**Affiliations:** 1 College of Science and Technology, Ningbo University, 521 Wenwei Road, Ningbo 315300, China; 2 Department of Smart Fab. Technology, Sungkyunkwan University, Suwon 16419, Republic of Korea

**Keywords:** cellulose hydrogels, biocompatibility, sustainable materials, preparation techniques, application

## Abstract

Cellulose hydrogels, formed either through physical or chemical cross-linking into a three-dimensional network from cellulose or its derivatives, are renowned for their exceptional water absorption capacities and biocompatibility. Rising demands for sustainable materials have spurred interest in cellulose hydrogels, attributed to their abundant supply, biodegradability, and non-toxic nature. These properties highlight their extensive potential across various sectors including biomedicine, the food industry, and environmental protection. Cellulose hydrogels are particularly advantageous in applications such as drug delivery, wound dressing, and water treatment. Recent large-scale studies have advanced our understanding of cellulose preparation and its applications. This review delves into the fundamental concepts, preparation techniques, and current applications of cellulose hydrogels in diverse fields. It also discusses the latest advances in nano-lignin-based hydrogels, providing a comprehensive overview of this promising material and offering insights and guidance for future research and development.

## 1. Introduction

Over recent decades, the field of materials science has undergone significant transformations, increasingly emphasizing sustainability and green chemistry [1,2,3,4,5]. This period has seen the extensive development of advanced and innovative nanomaterials, which have revolutionized traditional approaches across multiple industries and expanded the boundaries of previous research [6]. In this context, bio-based nanomaterials [7] have attracted considerable attention due to their renewability, strong biocompatibility, and exceptional biodegradability [8]. Among these, cellulose stands out as one of the most abundant natural polymers on Earth, playing a crucial role in driving sustainable technological advancements [9].

Cellulose, a linear polysaccharide composed of D-glucose units linked by β(1→4) glycosidic bonds, features anhydroglucose units endowed with active hydroxyl groups at the C2, C3, and C6 positions [10]. These groups exhibit significant reactivity, enabling cellulose to undergo diverse chemical modifications and to interact with various materials, thereby facilitating the formation of hydrogels [11]. The inherent properties of cellulose, such as its abundance, renewability, biocompatibility, and biodegradability, render it an exceptional substrate for hydrogel development, surpassing other biomaterials [12]. The molecular architecture of cellulose, characterized by reactive hydroxyl groups, is critical for hydrogel synthesis, enabling essential processes such as cross-linking and functionalization that tailor hydrogel properties to specific applications [13].

In recent years, cellulose hydrogels, a type of cellulose-based biomaterial, have emerged as a focal point of research [14,15,16,17]. These hydrogels consist of cellulose polymers, which derive their unique physical [18] and chemical [19] properties from the inherent structure of cellulose and the novel attributes imparted through advanced technological modifications. The preparation of cellulose hydrogels typically involves methods such as physical, chemical, and radiation crosslinking, each method distinctly influencing the hydrogel’s structural and functional characteristics [20]. The properties of these hydrogels are critically dependent on the size, shape, and texture of their internal nanostructures [21], which are optimized by precisely controlling the extent of integration within the polymer matrix [22]. Nanocellulosic materials, such as cellulose nanofibrils (CNFs) and cellulose nanocrystals (CNCs), have garnered widespread attention due to their exceptional mechanical properties, biodegradability, and versatility [23]. Cellulose nanofibrils are long, flexible fibers, typically several micrometers in length and a few nanometers in diameter, making them ideal for reinforcing hydrogels and enhancing their mechanical strength [24]. Additionally, the development and application of nanocellulose-based materials, particularly hybrid composites that incorporate nanocellulose polymers, have garnered extensive research interest [25,26,27,28,29]. These hybrid composites exhibit unique physical and chemical properties and broad biological functionality due to their natural polymer nanostructure, leading to a wide range of applications [30] such as personal care products [31], noble-metal sorption [32], bone regeneration self-cleaning textile [33], the food industry [34], electronics [35,36,37], energy harvesting [38], biomedicine [39,40,41,42], and environmental protection [43,44]. This underscores the versatile utility of nanocellulose-based materials in advancing sustainable solutions [45,46].

With increasing advanced research on cellulose hydrogels [47], the evolution of their preparation technology has become especially crucial [48]. Cellulose, which is rich in hydroxyl groups, possesses a unique chemical structure that provides an ideal foundation for developing hydrogels with distinctive structures and properties. Utilizing ultrasonic assistance generates fine bubbles and high-energy regions between cellulose molecules, enhancing the efficiency and uniformity of chemical reactions. This leads to the production of hydrogels with consistent quality and specialized functions [49]. The acid-treated mesh employs a mild acid to adjust the molecular weight and surface characteristics without compromising the main cellulose chain, thereby improving the formation capabilities and stability of the hydrogels. In situ polymerization within the cellulose matrix initiates a reaction that forms a densely cross-linked network, enhancing the mechanical strength and durability of the hydrogel [50]. Janus hybrid hydrogels broaden the application spectrum by incorporating materials with dual properties into the cellulose matrix, imparting unique interfacial activity and versatility [51]. Furthermore, 3D printing has revolutionized the flexibility and precision of hydrogel structure design, enabling the fabrication of customized and intricate hydrogel structures [52]. These advanced preparation techniques have not only significantly optimized the structural and functional properties of cellulose hydrogels but also expanded their potential applications across diverse fields such as drug delivery, tissue engineering, and environmental remediation.

Cellulose hydrogels has become a popular research focus, making it essential to summarize the latest advancements in cellulose hydrogels. This paper provides a comprehensive review of the basic concepts, advanced preparation techniques, and performance optimization methods of cellulose hydrogels, as well as their current applications and potential in various emerging fields. The review begins by defining cellulose hydrogels, highlighting their structural characteristics and how they differ from traditional hydrogels. It then proceeds to summarize innovations in cellulose hydrogel preparation techniques, including chemical, physical, and radiation cross-linking. These developments not only enhance the structural and functional attributes of the hydrogels but also expand their application prospects in environmental, biomedical, and industrial domains. The objective of this review is to offer valuable insights and guidance for future research and development in this promising area.

## 2. Basic Properties and Chemical Structure of Cellulose

Cellulose, a prevalent organic polymer, is abundantly found in nature and plays a pivotal role in numerous fields due to its unique physicochemical properties and biocompatibility. This section delves into the molecular structure and physicochemical characteristics of cellulose, providing a foundational understanding that is essential for exploring its applications in hydrogel preparation.

**Figure 1:**
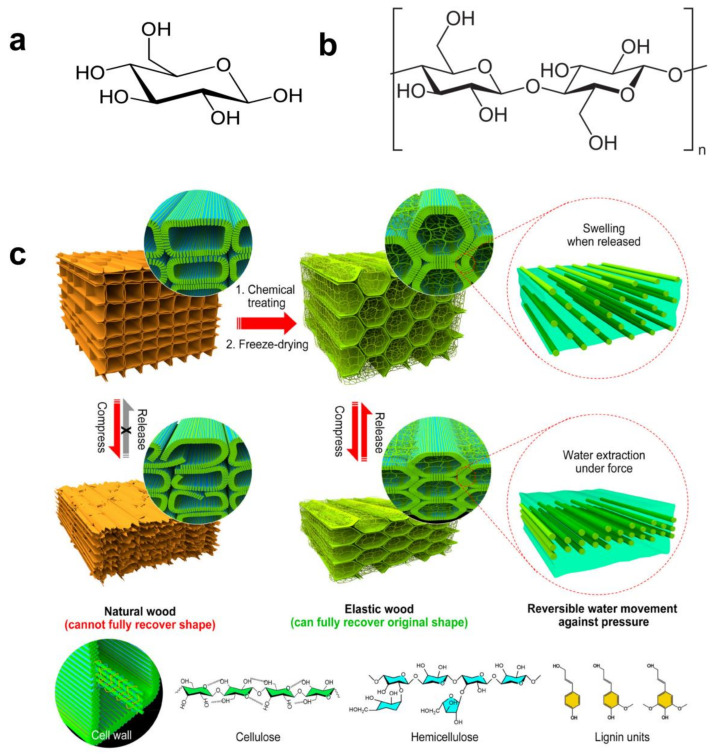
Chemical structures relevant to cellulose hydrogel: (**a**) the chemical structure of β-D-pyranoglucose, (**b**) the chemical structure of cellulose, and (**c**) a comparative diagram between natural wood and elastic wood (Reprinted with permission from the reference [56]. Copyright © 2020 American Chemical Society).

Cellulose consists of long-chain polysaccharides composed of β-D-glucose units (Figure 1a), interconnected through β-(1→4)-glycosidic bonds(Figure 1b). The presence of these β-glycosidic bonds makes cellulose insoluble in water [53], a property essential for its stability and utility. The fundamental repeating unit within cellulose molecules, known as cellobiose, forms linear polymer chains. These chains exhibit a dual structural arrangement: highly ordered crystalline regions and less-structured amorphous regions [54]. This organization is facilitated by hydrogen bonding and van der Waals forces, endowing cellulose with its distinctive mechanical strength and chemical stability [55].

The physicochemical properties of natural cellulose (Figure 1c) include renewability, biodegradability, non-toxicity, and chemical stability. These attributes collectively position cellulose as an ideal substrate for biomaterials preparation. However, the inherent high porosity of natural wood cellulose [57], as demonstrated by previous studies, contributes to brittleness and reduced resilience. Notably, the chemical modification of cellulose, especially targeting its hydroxyl groups, serves as a strategic approach to enhance its solubility, hydrophilicity, and biocompatibility [58].

An illustrative example of these modifications is the development of elastic memory wood, depicted in Figure 1c. This innovative material demonstrates the reversible movement of water [59] between the hydrophilic cellulose nanofiber-based cell walls and the gel-like substances within the lumens, thereby enhancing resilience and flexibility. Such chemical modifications underscore the adaptability of cellulose, tailoring its properties to meet specific application needs and significantly broadening its applications in biomaterial engineering and related fields.

## 3. Factors Effecting the Properties of Cellulose Hydrogels

This section delves into the environmental and procedural factors that critically influence the physical and chemical properties of cellulose hydrogels. These factors encompass a range of conditions, including pH levels, temperature settings, and the choice of solvents, which can significantly alter the cellulose’s solubility and the efficacy of the hydrogel’s crosslinking process. Understanding and controlling these variables is essential for tailoring the hydrogel’s functionality and stability, ensuring that they meet specific performance criteria required for various applications in fields such as biomedicine, environmental science, and materials engineering.

### 3.1. Solvent Selection and Cellulose Dissolution

Solvent selection is pivotal in the dissolution of cellulose, the initial step in cellulose hydrogel preparation. Due to its limited solubility [60] in most solvents, choosing effective solvent systems is crucial to enhancing cellulose solubility and consequently influencing the structure and properties of the resulting hydrogels [61]. Traditional solvents such as N-Methylmorpholine N-oxide (NMMO) ionic liquids [62,63], aqueous solutions containing alkali metal hydroxides [64,65], and LiOH/DMSO alkali/polar organic solvent systems have been extensively employed. There is also a growing interest in using green solvents, like deep eutectic solvents, which effectively dissolve cellulose while preserving its biocompatibility and biodegradability—key considerations in hydrogel preparation.

### 3.2. Swelling Kinetics, Temperature, and pH Effects

The formulation of cellulose hydrogels is significantly influenced by pH levels, temperature settings, and swelling kinetics [66]. Higher temperatures facilitate cellulose dissolution and speed up crosslinking reactions, creating a stable hydrogel network swiftly [67]. On the other hand, pH affects the cellulose’s ionization state, impacting its structural integrity and interactions within the hydrogel matrix. Swelling kinetics, which describe the hydrogel’s water absorption rate, are crucial for applications requiring rapid hydration, such as agricultural hydrogels [68] or medical dressings [48]. For example, in wound healing hydrogels, a slightly acidic pH (close to skin’s pH of 5.5) and lower temperatures help ensure bio-compatibility and sufficient mechanical strength [69]. Adjusting these factors optimizes hydrogel properties like mechanical strength, pore size, degradation rates, and swelling behavior, crucial for diverse applications from drug delivery to tissue scaffolds. Proper management of these parameters ensures the hydrogels meet specific needs, enhancing their functionality across various applications.

### 3.3. Crosslinking Methods

Crosslinking methods play a pivotal role in determining the structural and functional characteristics of hydrogels [70]. Table 1 presents a summary of cellulose hydrogel properties based on different preparation techniques. These methods, which include physical, chemical, and irradiation techniques, directly influence the network architecture of hydrogels, thereby affecting their mechanical strength, elasticity, and response to environmental stimuli [71]. For instance, chemical crosslinking often results in a more stable and rigid structure due to the formation of covalent bonds between polymer chains, which is ideal for applications requiring durability, such as implantable devices or load-bearing tissue scaffolds [72]. In contrast, physical crosslinking, which involves weaker, reversible bonds, allows for more flexible hydrogel networks that can respond dynamically to changes in pH or temperature, making them suitable for drug delivery systems where controlled release is necessary [73]. Irradiation crosslinking offers a unique advantage by allowing precise control over the crosslink density without the addition of chemical crosslinkers, reducing potential toxicity and making hydrogels safer for biomedical applications [74]. The choice of crosslinking method, therefore, must align with the intended application of the hydrogel, as it significantly affects the material’s performance and suitability for specific uses.

**Figure 2:**
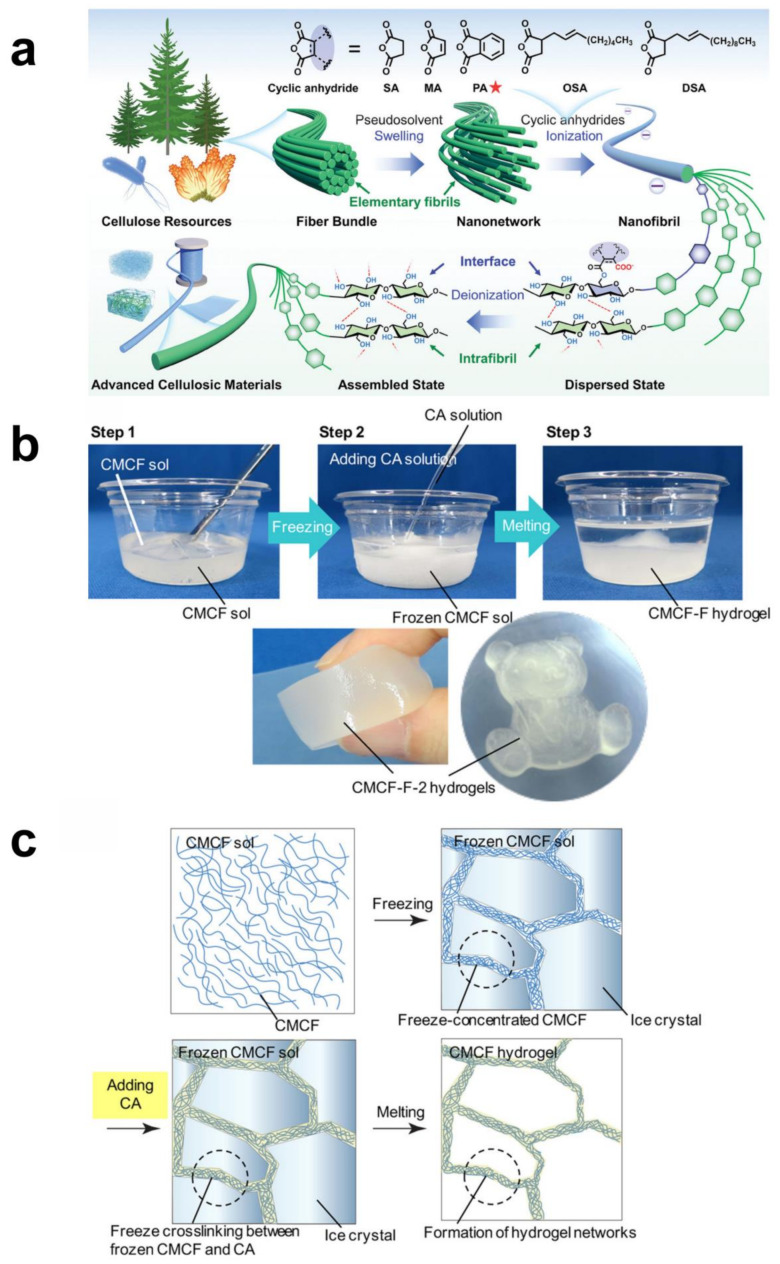
Preparation techniques for cellulose hydrogels: (**a**) solvent systems and cellulose dissolution, chemical cross-linking techniques, and exfoliation of cellulose elementary fibrils through a dual-step method, and sophisticated cellulose material assembly through surface deionization (Reprinted with permission from the reference [81]. Copyright © 2024 Wiley-VCH). (**b**,**c**) Physical cross-linking methods (Reprinted with permission from the reference [73]. Copyright © 2020 American Chemical Society).

Conventional Preparation Techniques for Cellulose Hydrogels:

The preparation of cellulose hydrogels represents a prominent research area in materials science, largely attributed to their distinctive properties and broad application potential. The preparation process employs a variety of techniques that address the specific challenges of cellulose solubilization and cross-linking. These techniques include the use of solvent systems for dissolving cellulose, along with diverse cross-linking methods such as chemical, physical, and radiation-based approaches. Figure 2a shows a novel surface engineering strategy is employed for extracting cellulose elementary fibrils from various cellulosic sources. This method entails a two-step process of swelling and esterification, utilizing alkali/DMSO systems as pseudosolvents [81]. This approach selectively disrupts hydrogen bonds and van der Waals interactions among the less-accessible fibril surfaces, forming highly swollen networks without dissolution or degradation. Subsequent esterification of exposed hydroxyl groups, facilitated by the addition of cyclic anhydrides, results in the production of cellulose nanofibers (CNFs) bearing carboxyl moieties. Importantly, these processes are executed concurrently in a one-pot reaction, which transforms cellulose pulp into a stable CNF suspension [82]. The stability of this suspension is attributed to the electrostatic repulsions among the CNFs, preventing agglomeration and ensuring their uniform dispersion throughout the suspension. This surface engineering strategy provides a promising route for the efficient extraction and utilization of cellulose nanofibers, with potential applications spanning materials science, biotechnology, and environmental engineering.

### 3.4. Chemical Cross-Linking Methods

Chemical cross-linking involves introducing cross-linkers, such as epoxides [83], isocyanates [84], or aldehydes [85], to form a stable three-dimensional network [86,87]. These cross-linkers react with the hydroxyl groups on cellulose molecular chains [88], forming stable covalent bonds [89], significantly enhancing the mechanical strength and stability of the resulting hydrogel.

Although chemically cross-linked cellulose hydrogels exhibit improved performance characteristics, the selection and concentration of cross-linkers are crucial. Excessive or inappropriate use of cross-linkers can lead to toxicity, potentially posing risks to biological organisms. For example, certain isocyanates are known for their cytotoxic effects, which can limit their application in biomedical fields. Thus, it is imperative to develop cross-linking methods that not only enhance mechanical properties but also ensure biocompatibility.

Current research in this area focuses on identifying new, less toxic, and environmentally friendly cross-linkers, as well as optimizing cross-linking conditions to maintain desired properties without compromising safety. Advances in this field promise to yield new materials that are robust, durable, and safe for diverse applications, including in medical and environmentally sustainable technologies.

### 3.5. Physical Cross-Linking Methods

Physical cross-linking methods are essential for stabilizing cellulose chains, primarily leveraging physical forces such as hydrogen bonding, hydrophobic interactions, or charge interactions, rather than forming covalent bonds [90]. Notable techniques include freeze–thawing [91], thermogelation [92], and ionic cross-linking [93], each contributing significantly to the structural integrity of cellulose-based materials. For example, the freeze–thawing process facilitates physical cross-linking among cellulose molecules via repeated cycles of freezing and thawing, which though generally result in lower mechanical strength compared to chemical cross-links, provide excellent biocompatibility and adjustability [94].

Moreover, scientists devised a freeze cross-linking technique to create a physically cross-linked hydrogel with significant compressive strength and recoverability, employing carboxymethyl cellulose nanofiber (CMCF) and citric acid (CA) (Figure 2b). The process of CMCF hydrogel formation entailed the addition of an aqueous solution of CA to a frozen CMCF sol, succeeded by thawing the sol [73]. The principle and procedure for preparation is presented in Figure 2c. This technique enhances the compressive strength and recoverability of the hydrogel [73]. Additionally, the hydrogel’s properties, including its efficacy as an adsorbent for removing toxic substances, have been thoroughly investigated.

Given the edibility and high biodegradability of CMCF and CA, the resultant hydrogels are considered non-toxic and environmentally friendly. Crucially, this method does not require complex procedures or synthetic reagents, utilizing only CMCF, CA, and water, thus promoting its practicality and sustainability.

### 3.6. Radiation Cross-Linking Methods

Radiation cross-linking [74] involves the use of γ-rays, electron beams [95], or ultraviolet light [96] to irradiate cellulose, thereby initiating free radical reactions that facilitate cross-linking. This technique enables the preparation of cellulose hydrogels without the need for chemical cross-linkers, thus avoiding potential toxicity concerns. Hydrogels produced via radiation cross-linking exhibit exceptional transparency, uniformity, and mechanical properties, making them particularly suitable for applications in 3D printing technology [97]. However, the efficacy of this method depends critically on the precise control of the radiation source, dosage, and exposure duration to ensure optimal performance and safety of the hydrogels.

The various preparation techniques for cellulose hydrogels each offer unique advantages and limitations [98,99]. The selection of the appropriate method is contingent upon the specific requirements of the intended application. As demands for environmental protection and biocompatibility increase, the development of more efficient and environmentally friendly preparation techniques remains a key focus of future research. Additionally, thorough investigations into different preparation methods can enhance the performance of cellulose hydrogels, broadening their application prospects in fields such as biomedicine, environmental protection, and beyond.

## 4. Performance Evaluation of Cellulose Hydrogels

The performance evaluation of cellulose hydrogels is crucial to ascertain their efficacy across diverse applications. This evaluation primarily encompasses assessments of mechanical properties, water absorption capabilities, and tests for biocompatibility and biodegradability.

### 4.1. Mechanical Properties of Cellulose Hydrogels

The mechanical properties of cellulose hydrogels are critical in a variety of applications [100,101,102], particularly in biomedical [103] and engineering [69] applications where materials must exhibit specific strengths and elasticity. Mechanical properties are evaluated using methods such as compression [104], tension, and shear characterization. These analyses provide crucial data on the strength [105], elastic modulus [106], elongation at break, and toughness of cellulose hydrogel. The factors such as cross-linking density, molecular weight distribution [107], and preparation methods influence the mechanical properties of cellulose hydrogel. Enhancements in mechanical performance are achieved by optimizing the degree of cross-linking and incorporating reinforcing fillers like nanoparticles or fibers.

**Figure 3:**
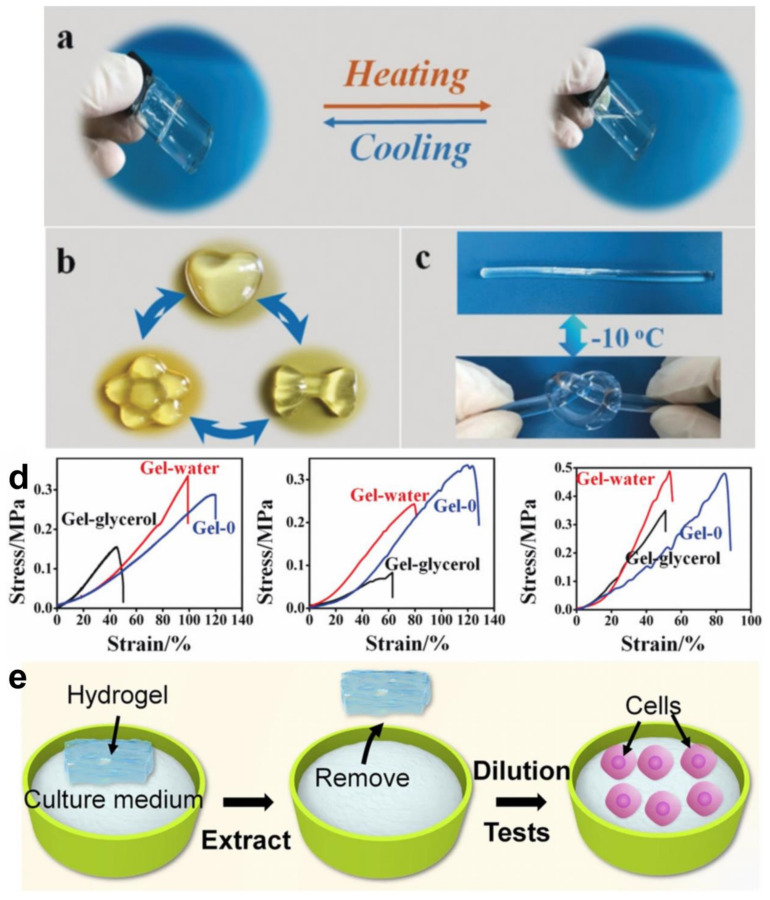
Performance evaluation of cellulose hydrogel and appearance of cellulose hydrogel: (**a**) photographs showing the thermal reversibility of gel glycerol, (**b**) photographs showing the remodelability of gel glycerol, and (**c**) an extended piece of gel glycerin exhibits good tensile properties at −10 °C, (**d**) tensile stress–strain curves of cellulose hydrogels at 25, −20, and −60 °C (Reprinted with permission from the reference [108]. Copyright © 2019 Wiley-VCH). (**e**) Cytocompatibility test of cellulose hydrogels [109].

Photographic evidence highlights the unique characteristics of cellulose hydrogels [108], such as thermal reversibility (Figure 3a) and remoldability (Figure 3b). The thermally reversible nature of these hydrogels is observed during heating-cooling cycles, allowing for easy modification of shape when molten cellulose is introduced into molds. Their remoldability originates from noncovalent cross-linking, primarily via hydrogen bonding and metal-cellulose interactions [108]. Additionally, the exceptional flexibility of the gel-glycerol system at −10 °C (Figure 3c) demonstrates its capability to withstand significant tensile deformation and recover its original shape effectively [108].

Further exploration of the tensile properties of cellulose gels at different temperatures (Figure 3d) provides deeper insights into the mechanical behavior of cellulose gels. The sol-gel poured into custom molds at room temperature to prepare the gel (Gel-0) and the sol-gel solidified using water as a reagent (Gel-water) exhibit varying tensile strength, elastic modulus, and elongation at break, highlighting the influence of water content on mechanical performance [108]. The increase in tensile strength with additional water is attributed to a higher cross-linking density, while a reduction in elongation at break indicates increased rigidity of the hydrogel network, likely due to water coordination with zinc ions. Despite the inherent mechanical limitations of physically cross-linked hydrogels, those composed of cellulose display sufficient mechanical adequacy for use in applications such as soft tissue replacements, thanks to their dense network of cross-links.

### 4.2. Water Absorption Performance

Water absorption performance is a critical parameter for cellulose hydrogels, particularly when utilized as moisture-absorbing materials or in drug delivery systems [110,111,112,113,114]. This performance is influenced by factors such as cross-linking density [115], pore structure [116], and the hydrophilicity of the material [116], influencing the water absorption performance, typically measured by water absorption rate [117] and water content [118], which reflect the gel’s ability to absorb and retain moisture. By adjusting preparation conditions, such as the type and concentration of cross-linking agents, the water absorption performance of cellulose hydrogels can be optimized to meet specific application requirements.

### 4.3. Biocompatibility and Biodegradability

Biocompatibility [119] and biodegradability [120] are crucial performance metrics for cellulose hydrogels in biomedical applications. Biocompatibility is defined as the capacity of a substance to interact with the biological body without inducing adverse reactions or toxicity. This characteristic is typically evaluated through cytotoxicity tests, blood compatibility tests, and animal experiments to ensure both safety and effectiveness (Figure 3e). Cellulose, as a natural polymer, generally exhibits good biocompatibility [121]. Biodegradability refers to the capacity of a material to decompose into low-molecular-weight compounds, either within the body or in the natural environment, which is vital for minimizing environmental impact and avoiding long-term negative effects in the body. The biodegradability of cellulose hydrogels is influenced by factors such as chemical structure, cross-linking density, and external environmental conditions like enzymes, microorganisms, and pH [122,123]. By choosing suitable preparation methods and conditions, the biodegradation rate of hydrogels can be controlled to align with specific application needs.

Evaluating the performance of cellulose hydrogels is vital to ensure their efficacy and safety in various applications. With continuous research and development, the creation of new hydrogel materials with enhanced properties is expected to meet the diverse needs of different fields.

### 4.4. Thermal Properties

The thermal properties of cellulose hydrogels are pivotal in determining their applicability across various environmental and biomedical settings [124]. These properties primarily include thermal stability and thermal conductivity [108]. Thermal stability assesses the ability of the hydrogel to maintain its chemical structure and mechanical integrity under elevated temperatures, which is crucial for applications that involve sterilization processes or outdoor usage [125]. Thermal conductivity impacts how effectively a hydrogel can distribute or resist heat, an important factor in therapeutic applications where temperature modulation is necessary for pain relief or inflammation reduction [126]. Analyzing these properties involves detailed testing to determine the hydrogel’s melting point, glass transition temperature, and thermal degradation thresholds [127]. This ensures that cellulose hydrogels perform reliably under different thermal conditions, whether utilized in controlled drug delivery systems or as insulating materials in construction.

## 5. Application Fields of Cellulose Hydrogels

### 5.1. Applications of Cellulose Hydrogels in Medical and Drug Delivery Fields

Cellulose hydrogels exhibit significant potential as drug delivery systems and wound healing materials in the medical field, primarily due to their excellent biocompatibility which minimizes systemic side effects [128,129]. In tissue engineering, cellulose hydrogels are utilized as scaffolding materials that enhance cell attachment and proliferation, thereby accelerating tissue regeneration. As wound dressing materials, they help maintain wound moisture, absorb exudates, and facilitate effective gas exchange, all of which contribute to faster healing processes.

**Figure 4:**
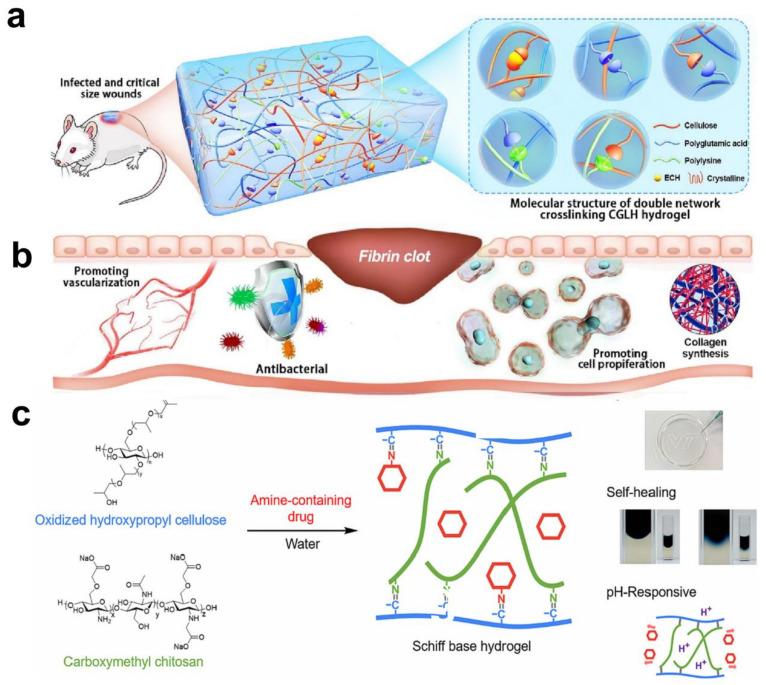
Cellulose hydrogels in medical and drug delivery fields: (**a**) Schematic illustration depicting the method of preparation and biomedical utilization of double-network CGLH. (**b**) CGLH exhibits potential in accelerating wound healing by eliminating bacteria and promoting vascularization, cell proliferation, and collagen deposition (Reprinted with permission from the reference [130]. Copyright © 2023 Elsevier). (**c**) Cellulose-based hydrogels are pH responsive to targeted drug delivery (Reprinted with permission from the reference [131]. Copyright © 2023 Elsevier).

An ε-PL-modified cellulose/γ-PGA hydrogel (CGLH) was developed employing a double-network strategy. The researchers first synthesized CGH through chemical crosslinking, followed by integration of ε-PL to enhance the hydrogel’s properties. The resulting molecular structure of the double-network CGLH is prominently displayed, showcasing the successful integration of the components (Figure 4a) [130]. Subsequently, extensive investigations were conducted to assess the biocompatibility and antibacterial properties of CGLH across various preclinical models [130]. The hypothesis was posited that CGLH could potentially expedite the healing process of infected and critical-size wounds by eradicating bacteria and facilitating crucial physiological processes like collagen deposition, vascularization, and cell proliferation (Figure 4b). The findings of this study suggest promising prospects for clinical translation of the developed hydrogel products. A pH-responsive, injectable, and self-healing hydrogel, crafted from oxidized hydroxypropyl cellulose (Ox-HPC) and carboxymethyl chitosan (CMCS), utilizes reversible imine bonds for its functionality (Figure 4c) [131]. This dynamic hydrogel shows enhanced drug release in slightly acidic conditions like those around tumor cells, demonstrating its potential for targeted drug delivery.

### 5.2. Applications of Cellulose Hydrogels in Environmental Engineering

**Figure 5:**
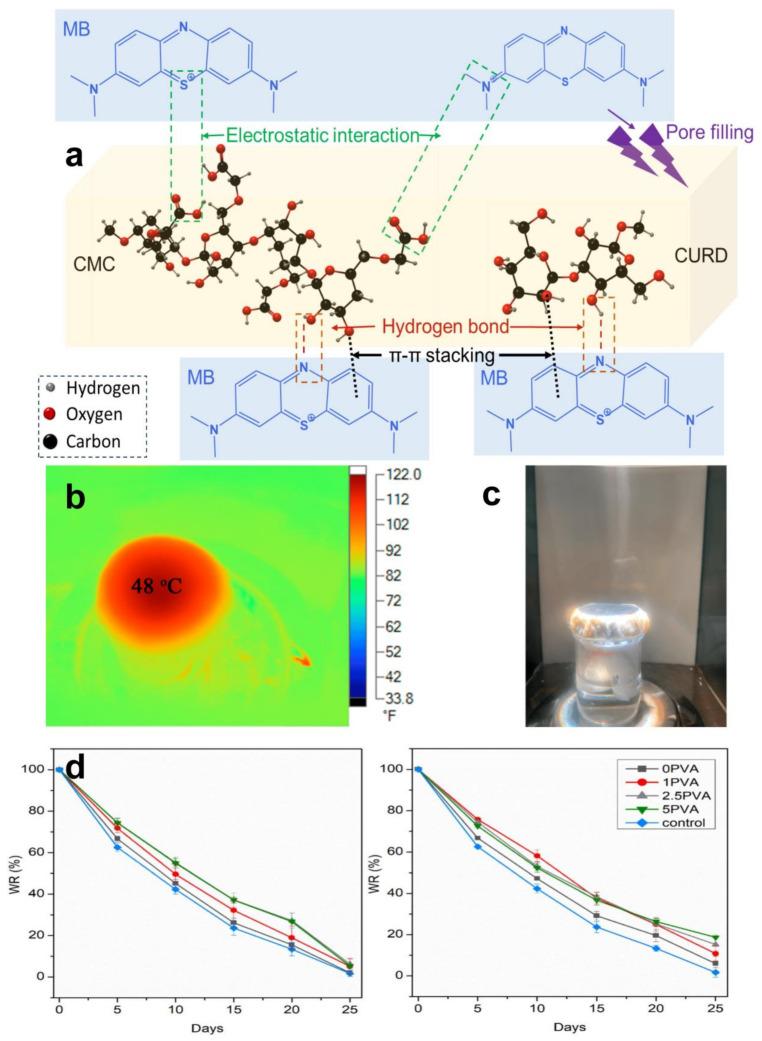
Cellulose hydrogels in environmental engineering fields. (**a**) A biodegradable solution to remove organic dyes from water via the mechanism of adsorption of MB onto CURD/CMC adsorbent (Reprinted with permission from the reference [153]. Copyright © 2024 Elsevier). (**b**,**c**) Picture of practical application of water vapor evaporation of composite materials under sunlight irradiation [140]. (**d**) Soil water retention experiment: comparison of swelling properties of hydrogel particle size ≥ 2 mm and hydrogel particle size ≤ 2 mm in soil (Reprinted with permission from the reference [68]. Copyright © 2024 Elsevier).

Cellulose hydrogels are increasingly utilized in environmental engineering as effective adsorbents for removing heavy metals and organic pollutants from water. These hydrogels facilitate pollutant adsorption through various mechanisms including electrostatic interactions, ion exchange, and hydrophobic interactions [132,133,134]. For example, carboxymethyl cellulose nanocrystalline hydrogels bind lead ions effectively through electrostatic adsorption (Figure 5a), while high-charge-density cationic hydrogels are used to treat anionic pollutants in wastewater [135,136,137,138,139]. Furthermore, innovative ultrafast photochromic materials, created by embedding tungsten oxide nanodots into cellulose fibers, exhibit exceptional photothermal conversion efficiency. This capability is particularly useful in solar-driven water evaporation experiments (Figure 5b,c), supporting the development of solar-powered seawater desalination technologies [140].

Cellulose hydrogels also play a crucial role in removing dyes such as methylene blue from wastewater, utilizing physical adsorption, ion exchange, or electron-sharing mechanisms for purification. Nanocomposite hydrogels, such as those made from carboxymethyl cellulose and polyacrylic acid, enhance adsorption efficiency by increasing their specific surface area [141,142,143,144,145,146,147]. In soil remediation, cellulose hydrogels improve soil quality by retaining moisture and reducing the leaching of harmful substances (Figure 5d). These hydrogels are also employed in encapsulating fertilizers, thereby improving fertilizer efficiency and supporting sustainable agricultural practices [148,149,150,151].

Cellulose hydrogels are applied in air purification to effectively adsorb particulate matter and toxic gases, improving air quality. Through these versatile applications, cellulose hydrogels demonstrate their immense potential as sustainable materials in environmental engineering [152].

### 5.3. Applications of Cellulose Hydrogels in Food Industry

**Figure 6:**
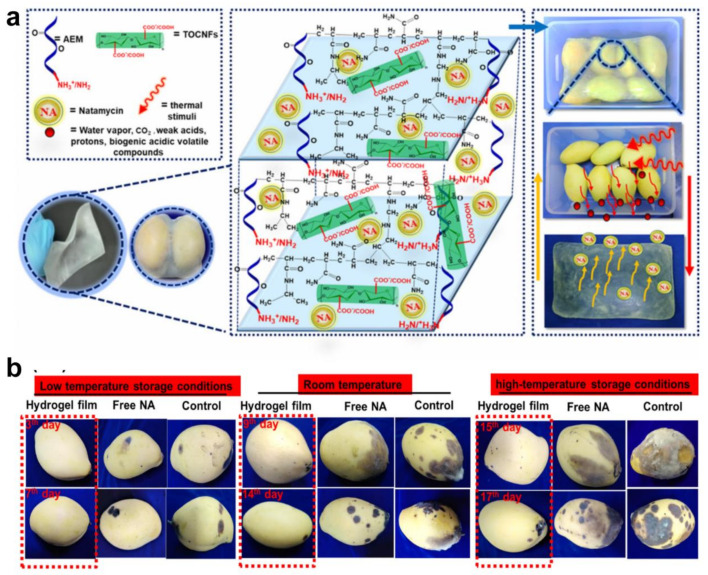
Cellulose hydrogels in environmental food industry. (**a**) Thermo-/pH-responsive preservative delivery based on TEMPO cellulose nanofiber/cationic copolymer hydrogel film in fruit packaging, and (**b**) the appearance development of mangos from two cultivars, subjected to different in vivo treatments and stored under the aforementioned conditions (Reprinted with permission from the reference [155]. Copyright © 2021 Elsevier).

In the food industry, cellulose hydrogels are primarily used as food additives and packaging materials [154]. As food additives, they serve as stabilizers and thickeners, enhancing the texture and stability of food (Figure 6a). The study examines the growth kinetics of escherichia coli and staphylococcus aureus against hydrogel films with 2,2,6,6-Tetramethylpiperidine-1-oxyl(TEMPO)-oxidized cellulose nanofiber/cationic copolymer, showing a moderate bacterial colony inhibition of around 40%. The hydrogel films displayed significant antifungal efficiency against aspergillus niger, particularly TEMPO-oxidized nanocellulose/cationic poly(N-isopropylacrylamide-co-acrylamide) (^NA^TOCNF/CP_NIPAM-AM_), which completely inhibited growth, demonstrating the potential of naringenin(NA) integrated into hydrogel films for enhanced antimicrobial effectiveness under acidic and/or high-temperature conditions, optimizing naringenin release rates and reducing naringenin concentration on fruit surfaces by 25–50% (Figure 6b) [155].

In the realm of food packaging, cellulose hydrogel-based biodegradable materials not only effectively extend the shelf life of food but also reduce environmental pollution. Lian et al. utilized cotton linter pulp as a cellulose source dispersed in ZnCl_2_/CaCl_2_ hydrates to form ionically cross-linked hydrogels, which were regenerated through ethanol exchange, offering a promising alternative to sustainably replace petroleum-based plastics with low biotoxicity [156]. Additionally, TEMPO-oxidized nanofibrillated cellulose from wheat straw was employed to synthesize pH/thermal-responsive hydrogel films of TEMPO-oxidized nanocellulose/cationic poly(N-isopropylacrylamide-co-acrylamide)(^NA^TOCNF/_CPNIPAM-AM_) through cationic modification with poly(N-isopropylacrylamide-acrylamide) and natamycin, establishing a novel dual stimuli-responsive preservative delivery system sensitive to climacteric fruit environmental cues using a naringenin preservative model. Ding et al. successfully constructed a pH-responsive RC (resin/cotton) thin film using cotton fabric, PVA, and acid-sensitive colorants. Under alkaline conditions, the film exhibits a color change in response to pH and demonstrates excellent leak-proof properties in acidic and alkaline environments. This preparation utilize for naked-eye detection of ethanol/cellulose-based pH sensors, applicable for monitoring ammonia generation and indicating the freshness of crustacean products [157].

### 5.4. Personal Care Products

Due to its excellent water absorption and non-irritating properties, cellulose hydrogel is widely used in personal care products like sanitary pads, diapers, and wet wipes [158]. Sodium alginate (SA) and cellulose nanocrystals (CNC) have been ingeniously combined to form cross-linked and semi-interpenetrating network hydrogels (SAH) with exceptional absorbency and mechanical properties, ideal for the core of adult incontinence pads [159]. Sodium carboxymethylcellulose (NaCMC) is mixed with starch and subjected to cross-linking using a distinctive blend of sodium trimetaphosphate (STMP) and aluminum sulfate (AlS). Through a process of phase inversion (NaCMC-PI) followed by freeze-drying, membranes composed of NaCMC/Starch with ideal cross-linking are produced to hinder dissolution and breakdown.

**Figure 7:**
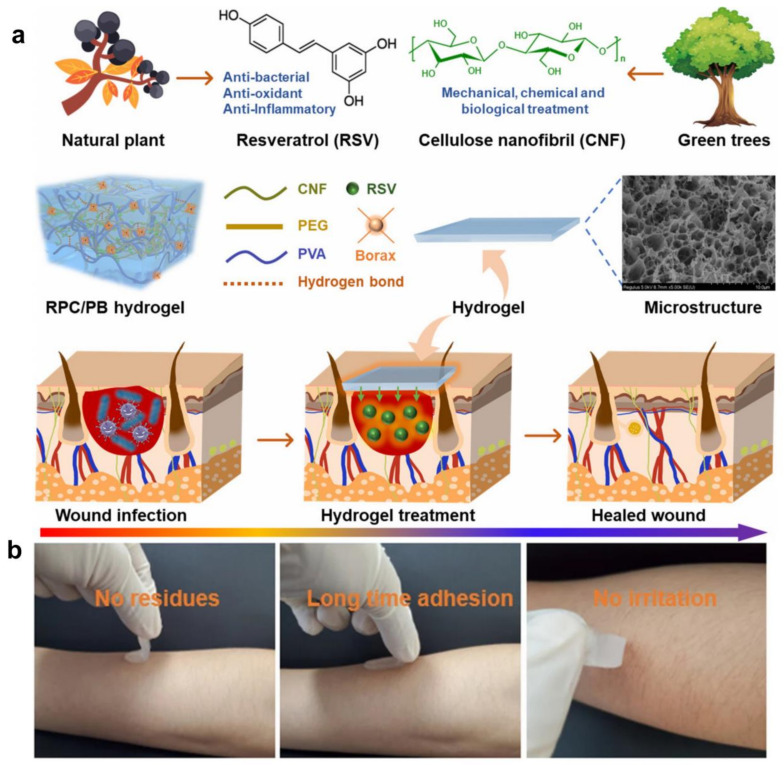
(**a**) A cellulose nanofibril-reinforced hydrogel, and the application of the hydrogel in wound healing; (**b**) RPC/PB-0.5: the hydrogel is removed from human skin without residue [163].

These environmentally friendly membranes feature a microtextured surface with excellent water and blood absorption capabilities, retaining around 50% of water. The provide an eco-friendly substitute for non-biodegradable polyacrylate hygiene items. In these products, it acts as an absorbent layer [160], quickly absorbing and locking in liquids to keep the skin dry and comfortable [161]. Additionally, cellulose hydrogel is used in cosmetics as a moisturizer and carrier, enhancing the performance and user experience of cosmetic products [162]. Furthermore, researchers have developed a novel cellulose hydrogel with robust mechanical, self-healing, pH-responsive, and antibacterial properties, making it suitable for wound dressing applications. The hydrogels are constructed using TEMPO-oxidized cellulose nanofibers (CNFs) and polyvinyl alcohol (PVA) as the framework, combining the distinct and complementary features of both materials (Figure 7a). Within this hydrogel matrix, PVA serves as the primary polymer network structure due to its remarkable elasticity and biocompatibility, while CNFs act as nanofillers to further enhance mechanical strength. Moreover, to confer antibacterial and antioxidant properties to the hydrogel, CNFs are functionalized with the natural antibiotic resveratrol (RSV) using polyethylene glycol (PEG) as a linker prior to hydrogel formation. This integration of materials not only enhances mechanical properties but also introduces bioactive functionalities, positioning the hydrogel as a promising candidate for advanced wound dressing applications. The explored the adhesive properties of cellulose hydrogels to human skin, revealing their excellent adhesion without causing harm to the skin even after prolonged use (Figure 7b). These findings underscore the multifaceted applications of cellulose hydrogels in medical and drug delivery fields, highlighting their potential as safe and effective materials for various biomedical applications.

As summarized in Table 2 overview of cellulose hydrogel compositions, applications, and performance outcomes, the diverse applications of cellulose hydrogels across various fields, from healthcare and environmental protection to the food industry and personal care, highlight their broad potential due to their unique properties.

## 6. Challenges and Future Directions

Despite the broad application prospects of cellulose hydrogels, their development faces several challenges. The high dependency on solvents, many of which are environmentally harmful, complicates large-scale production without damaging the environment. Additionally, the mechanical performance and stability of cellulose hydrogels often fail to meet the needs of specific applications, such as those requiring high material strength and biological stability in the medical field. Moreover, the biodegradation rate and pattern of cellulose hydrogels are difficult to precisely control, which may lead to performance degradation or unintended biological interactions in some applications.

### 6.1. In Vivo Performance and Long-Term Stability

Ensuring the in vivo performance and long-term stability of cellulose-based hydrogels remains a primary challenge. While these hydrogels exhibit excellent biocompatibility and biodegradability, their performance in biological environments can be unpredictable. Factors such as enzymatic degradation, immune response, and mechanical wear can affect their stability and functionality over time. Ensuring consistent performance in vivo requires comprehensive studies on the degradation behavior and biocompatibility of these hydrogels over extended periods.

### 6.2. Scalability and Cost-Effectiveness

Scalability and cost-effectiveness are also critical concerns. The processes used to produce cellulose-based hydrogels, especially those involving advanced modifications such as TEMPO oxidation or the incorporation of functional additives, can be complex and costly. Developing scalable manufacturing processes that maintain the quality and performance of these hydrogels while reducing costs is essential for their widespread adoption. This includes optimizing the use of raw materials, improving synthesis methods, and minimizing the need for expensive reagents or extensive purification steps.

### 6.3. Mechanical Properties

Achieving the desired balance between flexibility and robustness in cellulose-based hydrogels is another challenge. While physical and chemical cross-linking methods can enhance the mechanical strength of these hydrogels, ensuring they possess sufficient mechanical integrity to withstand physiological stresses without compromising their functionality remains difficult. This is particularly important for applications such as load-bearing materials or scaffolds for tissue engineering.

### 6.4. Environmental Impact

The environmental impact of hydrogel production and disposal is an important consideration. Although cellulose-based hydrogels are inherently biodegradable, the synthesis processes may involve the use of hazardous chemicals or generate waste products that need to be managed responsibly. Developing greener synthesis methods and ensuring that the entire lifecycle of the hydrogels, from production to degradation, is environmentally sustainable is crucial.

### 6.5. Functionalization and Customization

The functionalization and customization of cellulose-based hydrogels to meet specific application requirements also pose challenges. For instance, achieving precise control over the release profiles of encapsulated drugs or preservatives requires sophisticated design and engineering of the hydrogel matrix. Tailoring the physical and chemical properties of hydrogels for specific uses, such as responsive behavior to environmental stimuli, necessitates innovative approaches in material science and engineering.

### 6.6. Regulatory and Market Acceptance

Regulatory and market acceptance are critical hurdles. Ensuring that cellulose-based hydrogels meet the stringent regulatory standards for medical or food-related applications involves extensive testing and validation. Additionally, market acceptance depends on demonstrating the clear advantages of these hydrogels over existing materials and ensuring that they can be produced at a competitive cost.

Future research trends and directions will focus on the following:

### 6.7. Eco-Friendly Solvents and Green Preparation Technologies

Developing new eco-friendly solvents and dissolution processes to reduce reliance on harmful chemicals, aligning with green chemistry principles. Research on deep eutectic solvents, supercritical fluids, and ionic liquids will be emphasized.

### 6.8. Enhancing Mechanical Performance and Stability

Improving the mechanical properties and stability of cellulose hydrogels through optimized cross-linking strategies, nano-composite technologies, and molecular-level design. For instance, introducing nanocellulose or nanoparticles to reinforce the hydrogel structure or using advanced cross-linking methods to increase cross-link density and uniformity.

### 6.9. Smart and Responsive Hydrogels

Developing cellulose hydrogels with responsiveness to temperature, pH, and light to sensitively respond to environmental changes. These smart hydrogels have great potential in drug delivery, self-healing materials, and sensors.

### 6.10. Precise Control of Biocompatibility and Biodegradability

Precisely regulating the biocompatibility and biodegradation rate of cellulose hydrogels through chemical modification and bioengineering methods to meet the needs of different application scenarios.

Multifunctional integrated applications: combining cellulose hydrogels with other functional materials to develop new multifunctional integrated materials. For example, composite hydrogel systems integrating drug delivery, wound healing, and bio-detection.

### 6.11. Sustainable Production and Application

Strengthening the study of the life cycle of cellulose hydrogels, including raw material sourcing, production process, application lifespan, and final disposal, to promote their application under a sustainable development framework.

### 6.12. Multifunctional Integrated Applications

Combining cellulose hydrogels with other functional materials to develop new multifunctional integrated materials. For example, composite hydrogel systems integrating drug delivery, wound healing, and bio-detection.

Addressing these challenges will require a multidisciplinary approach, combining insights from materials science, engineering, biology, and environmental science. Continued research and development, along with collaboration between academia, industry, and regulatory bodies, will be essential to overcome these obstacles and unlock the full potential of cellulose-based hydrogels.

## Figures and Tables

**Table 1:** Summary of cellulose hydrogel properties based on different preparation techniques.

Method Category	Cross-Linking Mechanism	Specific Method	Physico-Chemical Properties	Ref.
Chemical Cross-Linking Methods	Microcrystalline	Hydrate Epichlorohydrin (ECH)	Water content: 76–84%, Mechanical strength: 21 ± 3 MPa, Fracture energy: 2.6 ± 0.4 MJ m^−3^	[14]
	Agarose Transition from Mono-Succinylation to Cross-Linking	Succinic Anhydride (SA)	Transparency: 89%, Strength: 815 g/cm^2^ Water content: 94.7%	[75]
	Formation of Ester Bonds between Two Polymer Chains	Citric Acid (CA)	Water content: 13.5–38.4%, Mechanical strength: 1.09 ± 0.11 MPa, Cell compatibility, Blood compatibility, and pH sensitivity	[69]
	Dual Cross-Linking of Nanocrystals	Gelatin Methacrylate (GelMA) and Ionically Cross-Linked Hyaluronic Acid (HA)	Porosity (>90%) and Average Pore Size: 130–296 μm Mechanical strength: 10 kPa, Enhancing tissue regeneration	[76]
Physical Cross-Linking Methods	Cellulose Nanocrystal Interface Adsorption and Hydrogen Bonding	Ultrasonication	Viscosity: 998.46 Pa.s Antioxidant	[77]
	Strong Hydrogen Bond Interaction	Freeze-Casting Method	Immobilized Papain pH, Thermal Stability, and Storage Stability	[78]
Radiation Cross-Linking Methods	CMC and Gelatin Cross-Linking	γ-Ray Radiation	Mechanical strength: 20–100 kPa Cell viability	[79]
	Glycosidic Bond Cleavage in Hydroxypropyl Methylcellulose Main Chain	Electron Beam Radiation	Temperature Sensitivity, Biodegradability	[80]

**Table 2:** Overview of cellulose hydrogel compositions, applications, and performance outcomes.

Types of Cellulose	Additives	Application	Characteristics	Ref.
Natural Cellulose	Magnesium Ion	Medical and Drug Delivery	Biocompatibility, antimicrobial efficacy, accelerated wound healing	[40]
Carboxymethyl Cellulose (CMC)	MXene	Environmental Engineering	Multifunctional conductive cellulose hydrogel	[36]
Nanocellulose	Alginate	Environmental Engineering	Enhanced moisture retention, antibacterial properties	[126]
Bacterial Cellulose (BC)	Silver Nanoparticles	Personal Care Products	Antibacterial effect, fast-reducing, anti-wrinkle and UV protection	[33]
Hydroxyethyl Cellulose (HEC)	Lignosulfonate	Environmental Engineering	High toughness and ductility, porous structure, dye absorption and removal	[164]
Exfoliated Fibrils	Proteins and polysaccharides	Food Industry	Recyclable, sustainable, economical	[154]

## Data Availability

The data that support the reviewed articles are contained within the article and more information are available from the referenced studies.
